# Supplementary material for: Association between acute respiratory acidosis and hyperkalemia during esophageal cancer surgery in the prone position: A multicenter retrospective observational study protocol
Source: PLoS One. 2026 Jan 27;21(1):e0337018. doi: 10.1371/journal.pone.0337018 (PMC12843540; doi:10.1371/journal.pone.0337018)
Supplement: Attachment — Submitted filename: Response to Reviewers.docx [file pone.0337018.s001.docx]

**Response to Reviewers**

**Manuscript ID:** [PONE-D-25-58930]

**Title:** Association between acute respiratory acidosis and hyperkalemia during esophageal cancer surgery in the prone position: A multicenter retrospective observational study protocol

We would like to thank the editor and the reviewer for their time and valuable comments. We have revised the manuscript in accordance with the suggestions, which have significantly improved the quality and clarity of our protocol. Our point-by-point responses are detailed below.

**Comment 1.** Defining the “intervention” time point: While selecting the lowest pH blood gas during the prone position is an effective and targeted strategy, pre-defining the criteria for data extraction is helpful. For example, should the lowest pH value be used initially, or should a minimum PaCO2 threshold (e.g., > 50 mmHg) be set to ensure that this time point truly represents a significant respiratory challenge? This would enhance consistency between the two centers.

**Response:** We appreciate this valuable suggestion regarding the definition of the intervention timepoint. As this is a retrospective study, our priority is to analyze the overall clinical picture without reducing the sample size unnecessarily; therefore, we have chosen not to apply a strict PaCO₂ threshold for the primary analysis. However, we agree that confirming the specific impact of significant respiratory acidosis is important.

To address this, we have added a sensitivity analysis restricted to patients with PaCO₂ ≥ 50 mmHg. This allows us to verify whether the association holds true in the population with strictly defined respiratory acidosis, as you suggested. We have updated the **Methods section (Secondary Outcomes)** accordingly.

**Comment 2.** Covariate selection for the multivariate model: To further strengthen the pre-description, consider listing candidate variables to be included in the model (e.g., ΔPaCO2, ΔLactate, baseline eGFR, fluid balance, norepinephrine dose, duration of prone position). Briefly describing the variable selection strategy (e.g., based on clinical relevance and/or univariate association with p-values < 0.1) will help further clarify the process.

**Response:** Thank you for this constructive comment. We agree that clarifying the variable selection process is essential.

As suggested, we have explicitly listed the candidate covariates in the **Methods** **section (Statistical Analysis)**. Regarding the variable selection strategy, we have adopted the approach based on clinical relevance and the sufficiency of outcome events to prevent overfitting, as mentioned in your comment.

**Comment 3.** Discussion of Confounding Factors: Briefly mention other perioperative factors that may affect potassium (such as the use of diuretics, insulin, or β2 agonists), and state that if the distribution is uneven, it will be recorded and considered as a covariate to further strengthen the causal inference.

**Response:** We agree with your insightful suggestion. Perioperative medications such as diuretics, insulin, and β2-agonists can indeed influence potassium levels.

Accordingly, we have added these medications to our data collection and will assess their association with the outcome in the univariate analysis. However, since our study population primarily consists of patients undergoing cancer resection who generally lack significant comorbidities (other than the malignancy), we anticipate that the number of patients using these medications may be very small.

If the number of cases is insufficient to include these variables in the multivariable model, we will report the descriptive data and address this as a limitation in the **Discussion section**. We have updated the **Methods** **section (Data Collection)** to reflect this addition.

**Comment 4.** Sample Size Justification: A minor supplement is to briefly explain why a difference of 0.5 mmol/L is considered clinically significant, or to link the increase in potassium to arrhythmic potentials by citing literature.

**Response:** We appreciate this opportunity to clarify our rationale. While the exact threshold at which an acute potassium increase becomes pro-arrhythmic is not definitively established, meta-analyses have demonstrated a U-shaped relationship between serum potassium levels and cardiovascular risk [Ref 5]. Moreover, recent studies emphasize that the magnitude of potassium change is a critical predictor of electrocardiographic abnormalities [Ref 6].

In the specific context of esophagectomy, which involves the prone position and surgical manipulation near the heart, we determined that capturing a 0.5 mmol/L increase is clinically meaningful to identify patients at potential risk. We have added this rationale and cited the relevant literature in the **Methods section (Sample Size Calculation)**.
